# Supplementary material for: An on-demand bioresorbable neurostimulator
Source: Nat Commun. 2023 Nov 11;14:7315. doi: 10.1038/s41467-023-42791-5 (PMC10640647; doi:10.1038/s41467-023-42791-5)
Supplement: Supplementary file 1 — Supplementary Information [file 41467_2023_42791_MOESM1_ESM.pdf]

Supplementary Information (SI)

An on-demand bioresorbable neurostimulator

Dong-Min Lee[1,2,10], Minki Kang[3,10], Inah Hyun[1,2,10], Byung-Joon Park[1,2], Hye Jin Kim[4], Soo Hyun Nam[5], Hong-Joon Yoon[6], Hanjun Ryu[7], Hyun-moon Park[8], Byung-Ok Choi[4,5,9], Sang-Woo Kim[1,2]

[1]Department of Materials Science and Engineering, Yonsei University, Seoul 03722, Republic of Korea

[2]Center for Human-oriented Triboelectric Energy Harvesting, Yonsei University, Seoul 03722, Republic of Korea

[3]School of Advanced Materials Science and Engineering, Sungkyunkwan University (SKKU), Suwon 16419, Republic of Korea

[4]Department of Neurology, Samsung Medical Center, Sungkyunkwan University School of Medicine, Seoul 06351, Republic of Korea

[5]Cell and Gene Therapy Institute (CGTI), Samsung Medical Center, Seoul 06351, Republic of Korea

[6]Department of Electronic Engineering, Gachon University, Seongnam 13120, Republic of Korea

[7]Department of Advanced Materials Engineering, Chung-Ang University, Anseong 17546, Republic of Korea

[8]Research and Development Center, Energy-Mining Co., LTD., Suwon 16226, Republic of Korea

[9]Samsung Advanced Institute for Health Sciences & Technology (SAIHST), Seoul 06351, Republic of Korea

[10]These authors contributed equally: Dong-Min Lee, Minki Kang, Inah Hyun

Correspondence should be addressed to B.-O.C. (e-mail: bochoi@skku.edu) and S.-W.K. (email: kimsw1@yonsei.ac.kr).

Contents

Supplementary Fig. 1 | Fabrication process of the on-demand bioresorbable neurostimulator.

Supplementary Fig. 2 | Images of the PHBV/PEG:ChCl membranes with different ChCl composition.

Supplementary Fig. 3 | Surface potential characterization of the PHBV/PEG:ChCl membranes.

Supplementary Fig. 4 | Underlying mechanism of attaining high surface potential along the ChCl addition.

Supplementary Fig. 5 | Material characterization of the PHBV/PEG:ChCl membranes.

Supplementary Fig. 6 | Electrical characterization of the PHBV/PEG:ChCl membranes.

Supplementary Fig. 7 | Acoustic transmission properties of a bare PHBV and a PHBV/PEG:ChCl membrane (thickness: 50 μm).

Supplementary Fig. 8 | Step-by-step mechanism of ultrasound-driven triboelectric energy generation.

Supplementary Fig. 9 | Simulated acoustic pressure field and displacement level of the triboelectric layer.

Supplementary Fig. 10 | In vitro experimental setup to measure ultrasound-driven triboelectric output performances of the ACT−TENG.

Supplementary Fig. 11 | Electrical characteristics of the ACT−TENG measured through the in vitro experimental setup.

Supplementary Fig. 12 | Field emission scanning electron microscope (FE-SEM) images of the PHBV/PEG:ChCl membranes.

Supplementary Fig. 13 | Underlying mechanism of the ultrasound-mediated transient process.

Supplementary Fig. 14 | Hydrolytic processes of PHBV and PEG.

Supplementary Fig. 15 | HIU-triggered transient performances of the PHBV and PHBV/PEG:ChCl membranes (20 mm width, 10 mm length, 50 μm thickness).

Supplementary Fig. 16 | Transient performances of PHBV and PHBV/PEG:ChCl without HIU-triggering events.

Supplementary Fig. 17 | Micro-CT images captured at the top and side views.

Supplementary Fig. 18 | Surgical procedure to implant the neurostimulator.

Supplementary Fig. 19 | Magnified view of the sciatic nerve.

Supplementary Fig. 20 | Experimental setup for the in vivo demonstration of peripheral nerve electrotherapy.

Supplementary Fig. 21 | Experimental setup for in vitro demonstration of the iPSC-driven motor neuron growth upon electrical impulses.

Supplementary Fig. 22 | Axon length plots after 72 h of culturing time upon different electric field intensities.

Supplementary Fig. 23 | Optical microscopic images of iPSC-driven motor neuron.

Supplementary Fig. 24 | In vivo electrical characterization of the ACT−TENG.

Supplementary Fig. 25 | Detailed procedure for preparing C22 mouse model.

Supplementary Fig. 26 | NCS results to evaluate the biosafety of the HIU to the sciatic nerve.

Supplementary Fig. 27 | Characterization of pH change upon the HIU treatment.

Supplementary Fig. 28 | Behavior analysis of the device-implanted mouse.

Supplementary Fig. 29 | Characterization of degradation time at different ultrasound intensities.

Supplementary Fig. 30 | Ultrasound-driven triboelectric output performances of ACT-TENG at various ultrasound intensities.

Supplementary Fig. 31 | The ACT-TENG was immersed in PBS (pH 7.4, 37 °C) for 7 days.

Supplementary Fig. 32 | XTT cell proliferation assay results.

Supplementary Note

Supplementary Note 1 | Fundamental background of the localization of acoustic pressure inside the PHBV encapsulation layer

Supplementary Note 2 | Macroscopic degradation mechanism of PHBV

Supplementary References

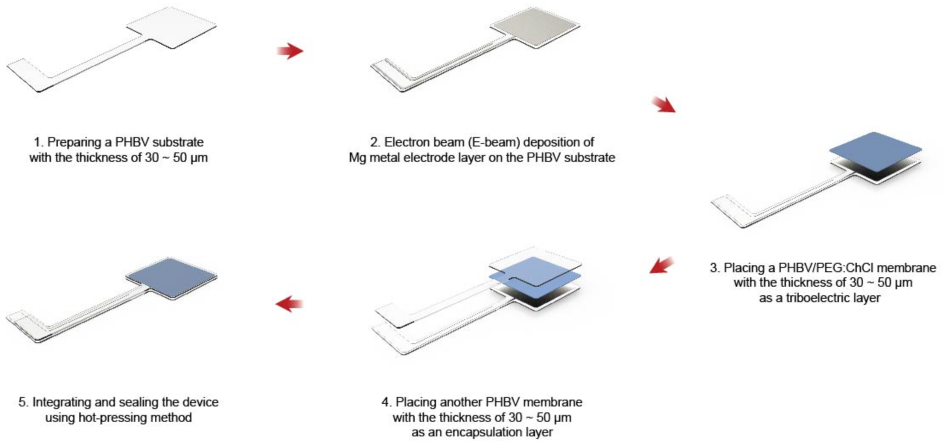

Supplementary Figure 1. **Fabrication process of the on-demand bioresorbable neurostimulator.** The thickness of the Mg electrode layer is 100 nm.

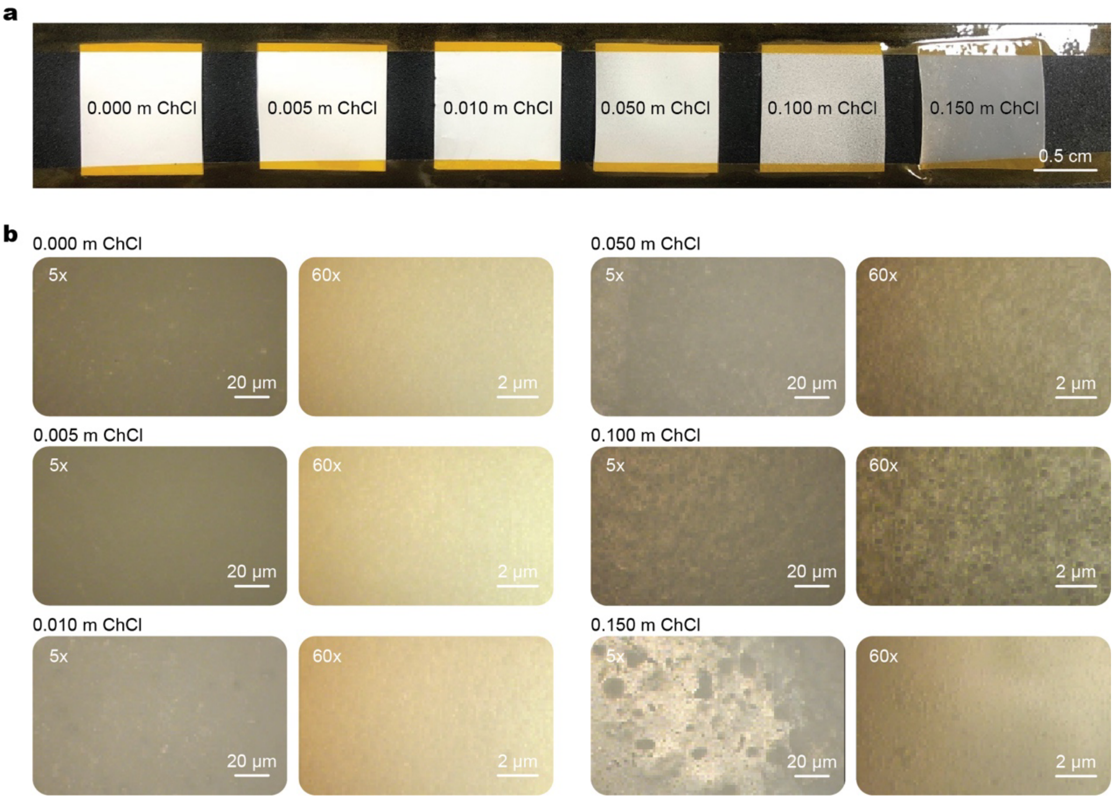

Supplementary Figure 2. **Images of the PHBV/PEG:ChCl membranes with different ChCl composition. a** Real image of the PHBV/PEG:ChCl membranes. **b** Optical microscopic images of the PHBV/PEG:ChCl membranes. The membranes with high ChCl concentration (≥ 0.100 m) exhibit high porosity that results in mechanical instability.

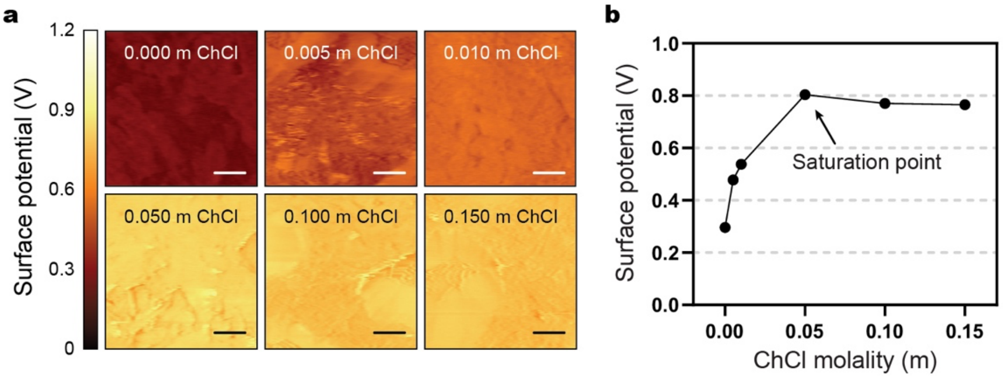

Supplementary Figure 3. **Surface potential characterization of the PHBV/PEG:ChCl membranes.** **a** Kelvin probe force microscopy (KPFM) imagery displaying the surface potential for each PHBV/PEG:ChCl membrane. The scale bars represent 1 µm. **b** The summation plot for the measured surface potential. The surface potential level reached the highest value at 0.05 m of ChCl concentration, and remained saturated at the higher ChCl molality.

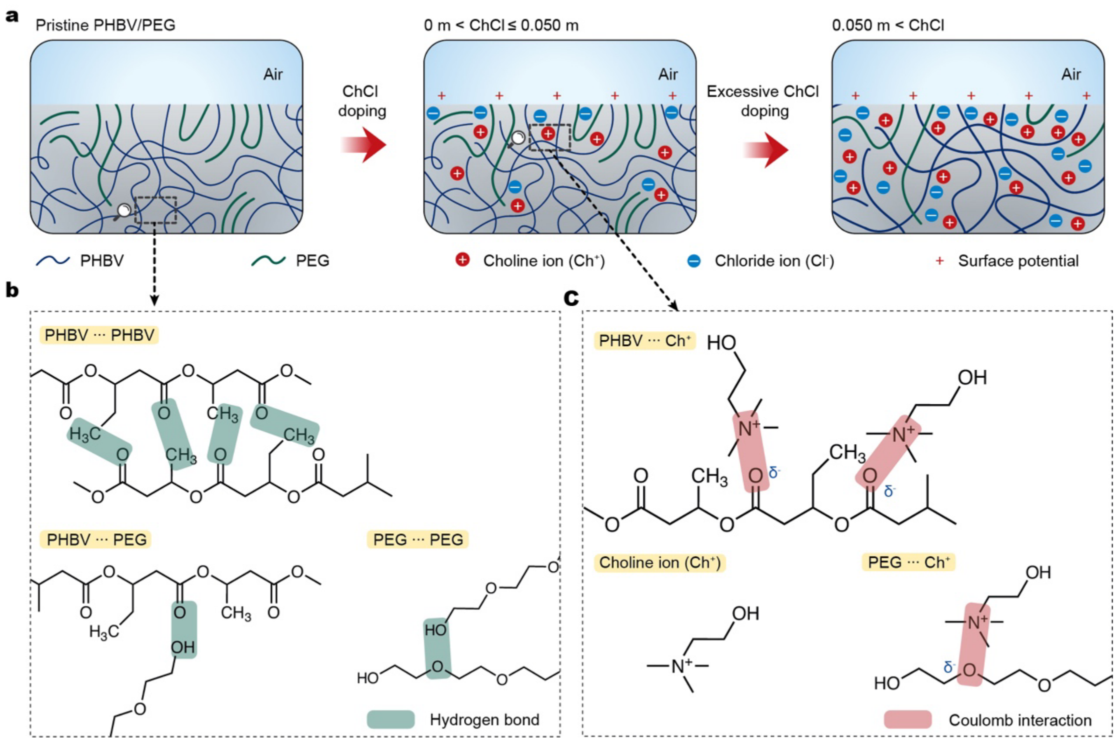

Supplementary Figure 4. **Underlying mechanism of attaining high surface potential along the ChCl addition. a** Schematic describing the change in the chain network of PHBV/PEG:ChCl along the ChCl addition. When ChCl ions are introduced, Ch^+ ions make intermolecular interactions with PHBV/PEG networks. Meanwhile, the mobile Cl^- ions are attracted to the surface to compensate the tribo-positive nature of the air[1]. Due to the presence of Cl^- ions at the surface, the PHBV/PEG/ChCl membrane exhibits improved surface potential. However, the excessive content of ChCl (more than 0.050 m) causes mechanical instability by mitigating the interactions between polymer chains. **b** Intermolecular interactions between the polymer chains. The chemical structure of PHBV is ascribed to the intermolecular interaction that involves a C−H···O hydrogen bond between the C=O group in one helical structure and the CH_3 group in the other helical structure[2]. **c** Coulombic interactions between Ch^+ ions and polymer chains.

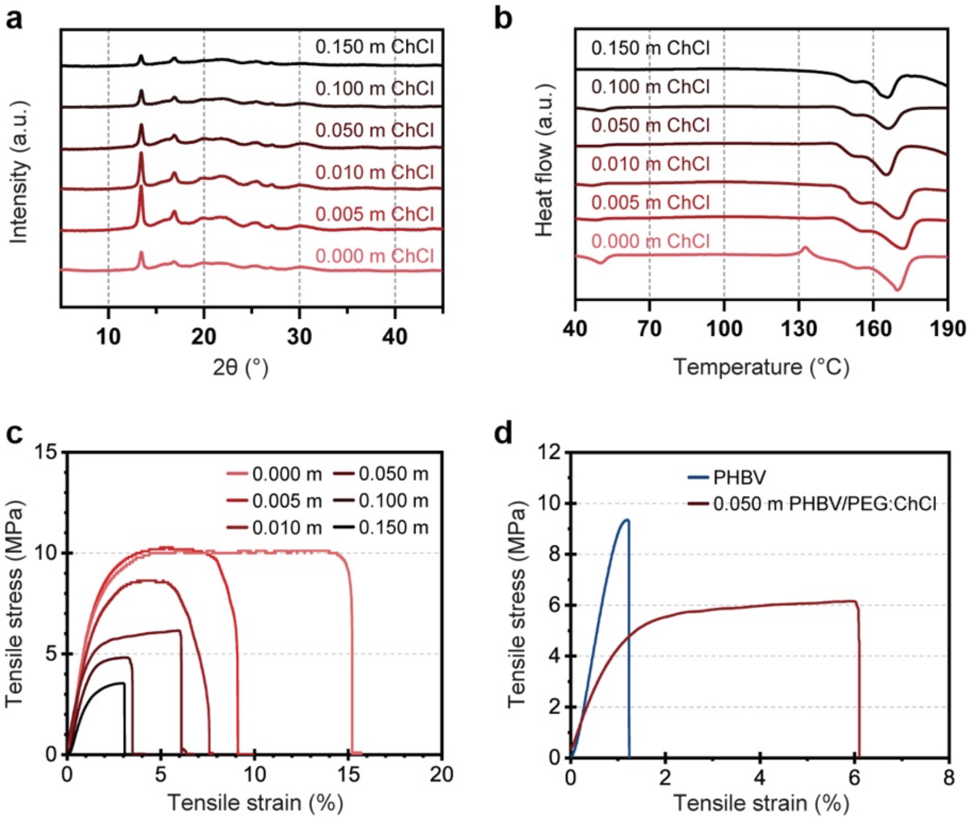

Supplementary Figure 5. **Material characterization of the PHBV/PEG:ChCl membranes.** **a** X-ray diffraction (XRD) patterns of the PHBV/PEG:ChCl membranes with different ChCl concentration. **b** Differential scanning calorimetry (DSC) patterns of the PHBV/PEG:ChCl membranes. The membranes with high ChCl concentration have lower crystallinity, implying their mechanical instability. **c** Stress–strain curves of the membranes, showing that the highly ChCl-doped composites are prone to plastic deformation with low resilience. **d** Comparison of mechanical characteristics between the bare PHBV and the optimized composite material.

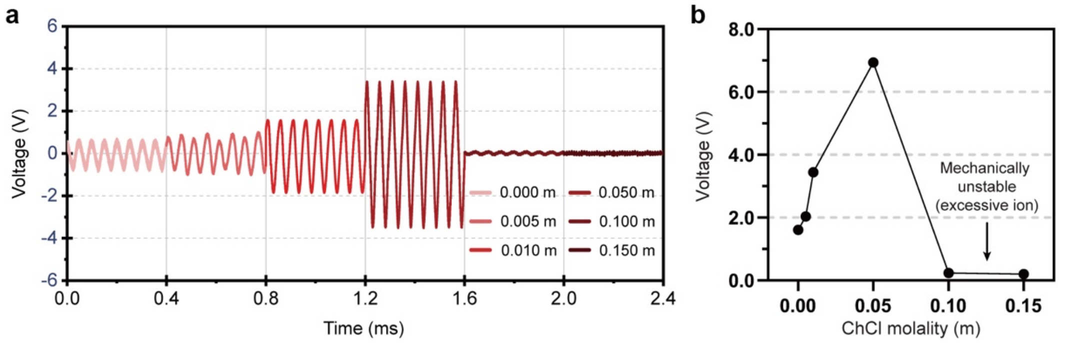

Supplementary Figure 6. **Electrical characterization of the PHBV/PEG:ChCl membranes.** **a** Measured triboelectric voltage at 40-megohm impedance by the ACT−TENG submerged into the water with 5 mm probe distance. **b** The summation plots of the triboelectric voltage output values. The ACT−TENGs with high ChCl concentration barely generate electrical output, due to the mechanical instability of the triboelectric layer.

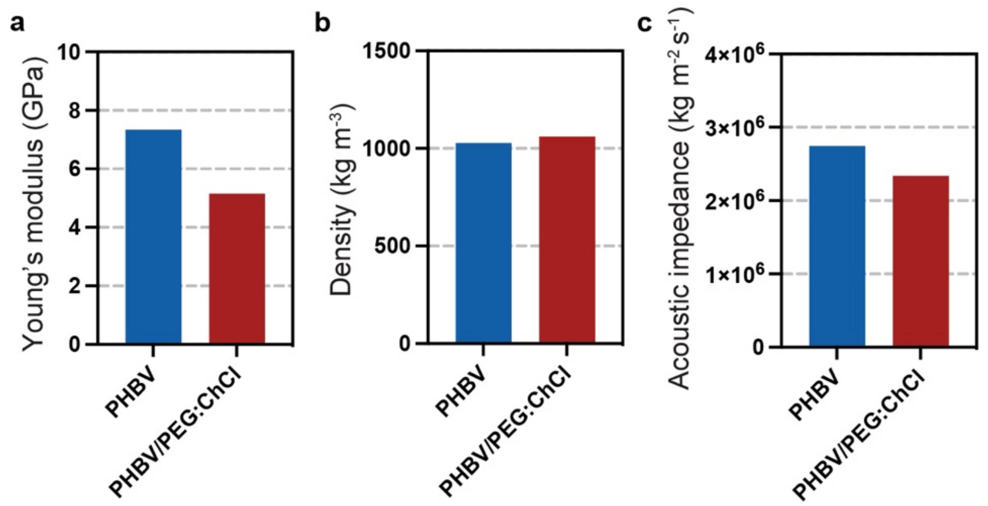

Supplementary Figure 7. **Acoustic transmission properties of a bare PHBV and a PHBV/PEG:ChCl membrane (thickness: 50 μm). a** Young's modulus value for each membrane. The values were obtained by the stress–strain curve shown in Supplementary Figure 5. **b** Measured density values using a specific gravity scale. **c** Calculated values of acoustic impedance derived from the Young's modulus and density.

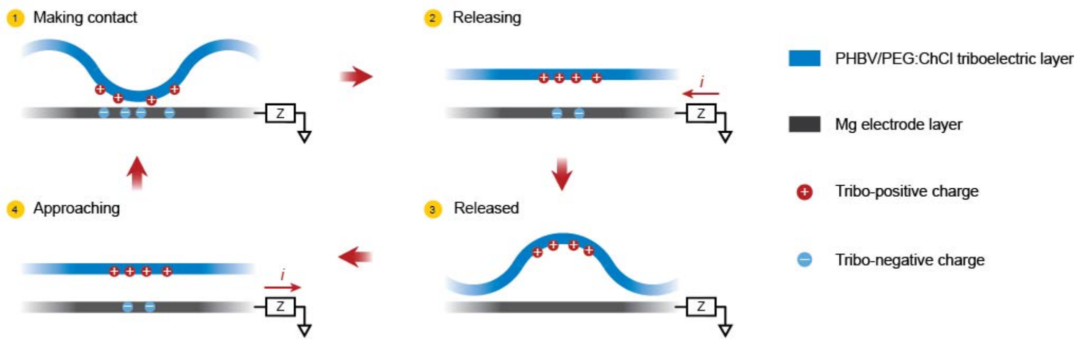

Supplementary Figure 8. **Step-by-step mechanism of ultrasound-driven triboelectric energy generation.** The ACT−TENG generates electrical potential based on the high frequency vibration of the PHBV/PEG:ChCl triboelectric layer.

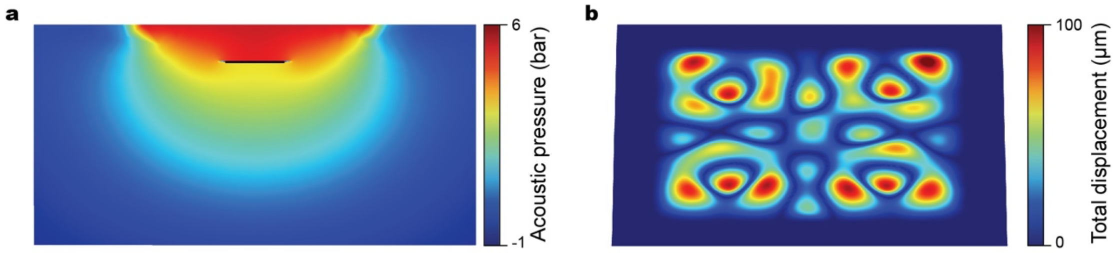

Supplementary Figure 9. **Simulated acoustic pressure field and displacement level of the triboelectric layer. a** Simulated acoustic pressure field upon applying a low-intensity ultrasound (20 kHz, 0.5 W cm^{-2}), confirming that the incident ultrasound was reflected by the 100 nm thick Mg electrode layer of the ACT−TENG. The diameter of the ultrasound probe is 3 cm. **b** Simulated displacement level of the PHBV/PEG:ChCl triboelectric layer (0.8 cm width, 0.8 cm length, 50 μm thickness) on the ACT−TENG.

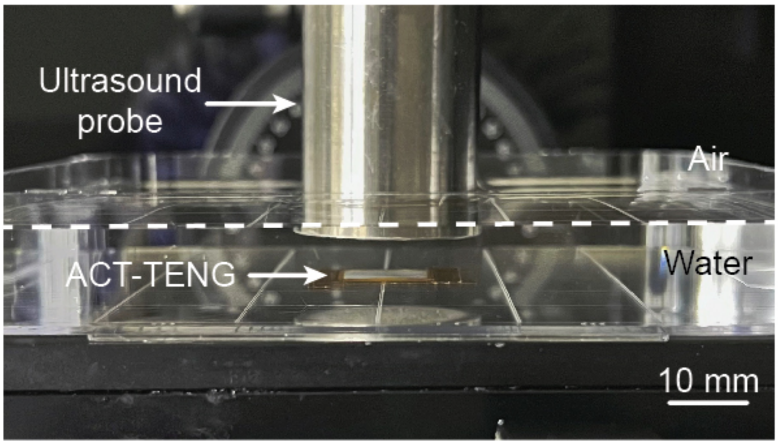

Supplementary Figure 10. **In vitro experimental setup to measure ultrasound-driven triboelectric output performances of the ACT−TENG.** The ACT−TENG was submerged into the deionized water with 5 mm probe distance.

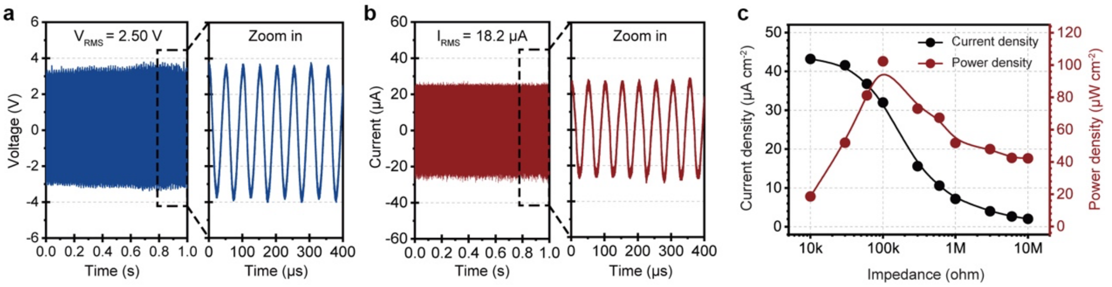

Supplementary Figure 11. **Electrical characteristics of the ACT−TENG measured through the in vitro experimental setup. a** Triboelectric voltage at 40 megohm impedance, and **b** current at 1 ohm impedance measured by the ACT−TENG. **c** Measured power and current density along the electrical impedance variation. The units of “k” and “M” refer to “kilo-” and “mega-”, respectively.

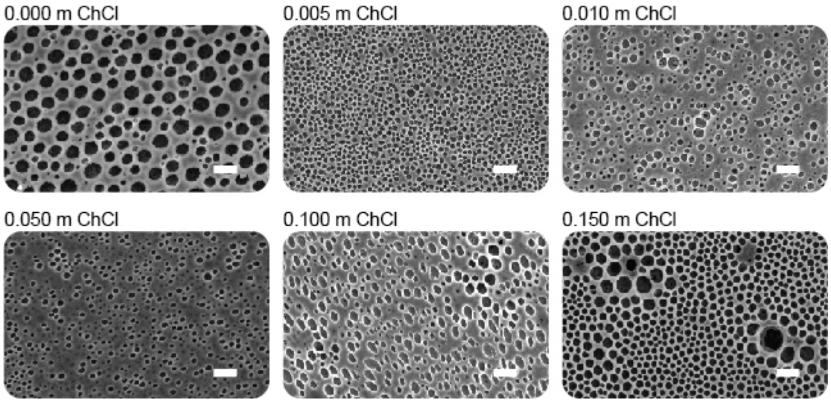

Supplementary Figure 12. **Field emission scanning electron microscope (FE-SEM) images of the PHBV/PEG:ChCl membranes.** The FE-SEM images support that the membranes exhibit highly porous microstructure, allowing for the localization of acoustic pressure inside their structure. The scale bars represent 20 μm.

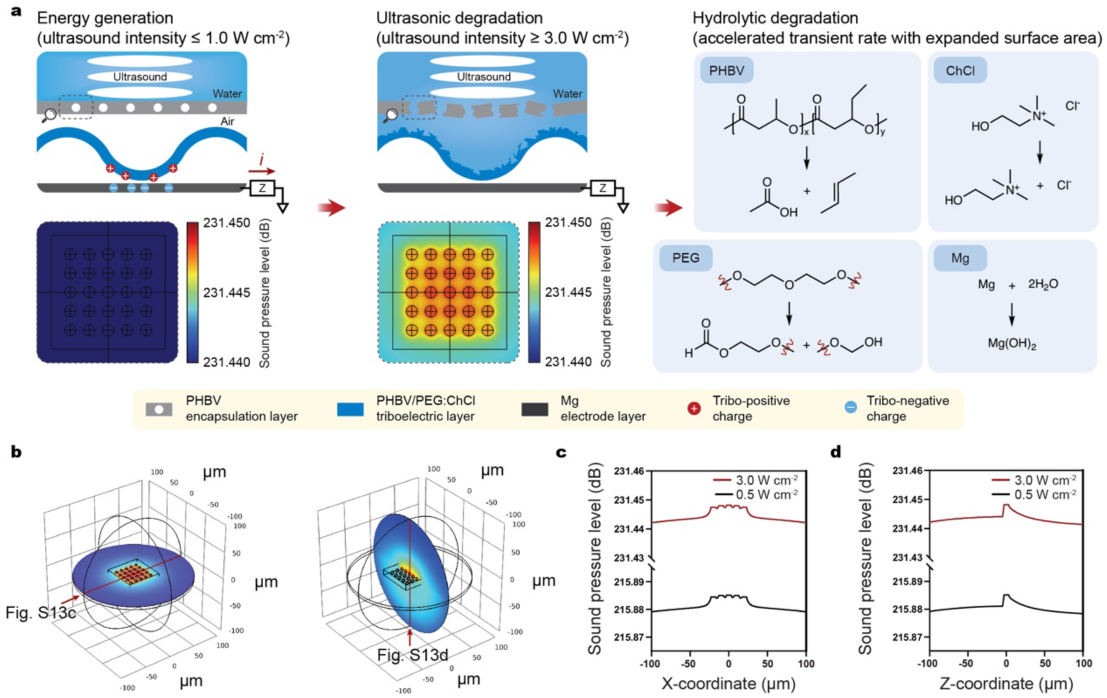

Supplementary Figure 13. **Underlying mechanism of the ultrasound-mediated transient process. a** Schematics describing the ultrasound-mediated transient process and simulated acoustic pressure field inside the PHBV encapsulation structure. The ACT−TENG generates reliable energy generating performances upon a low-intensity ultrasound (less than 1.0 W cm^{-2}), while it starts the biodegradation process upon HIU-driven triggering events (more than 3.0 W cm^{-2}). According to the findings detailed in Supplementary Note 1, it was observed that approximately 99.94 % of the acoustic wave experienced reflection at the interface of the PHBV and air, providing the localization of acoustic pressure within the pores of the PHBV membrane. To alleviate this localized acoustic pressure, mechanical disintegration is promoted in the vicinity of the PHBV porous structure. Notably, the PHBV within the inter-pore spaces is susceptible to acoustic pressure-induced mechanical stress, thereby facilitating material disintegration. The HIU-triggered disintegration results in the enlarged surface area of the constituent materials, which facilitates hydrolytic processes upon contact with biofluid. **b** 3D plotted simulation results of the acoustic pressure field. **c,d** The plotted acoustic pressure along the x-coordinates (**c**), and the z-coordinates (**d**), of a PHBV membrane.

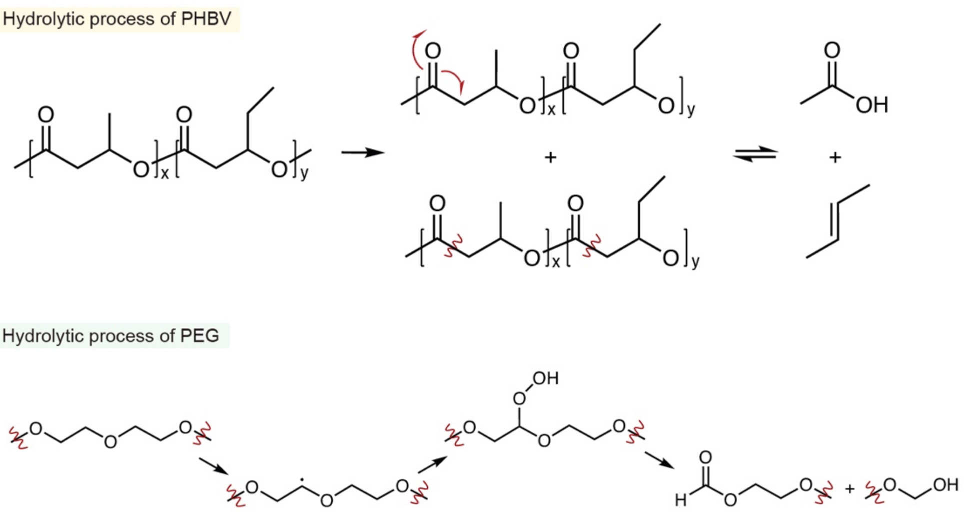

Supplementary Figure 14. **Hydrolytic processes of PHBV and PEG. Chemical formula showing the hydrolytic degradation of PHBV and PEG.**

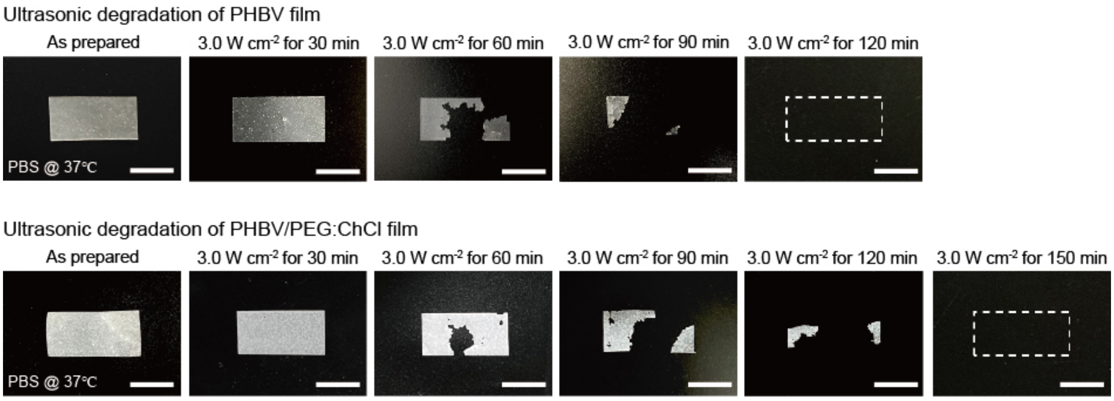

Supplementary Figure 15. **HIU-triggered transient performances of the PHBV and PHBV/PEG:ChCl membranes (20 mm width, 10 mm length, 50 μm thickness).** The PHBV and PHBV/PEG:ChCl membranes were piled up to mimic the ACT−TENG structure. The PHBV membrane was fully disintegrated in 120 min upon the HIU event, while the PHBV/PEG:ChCl membrane was eliminated in 150 min. The scale bars represent 10 mm.

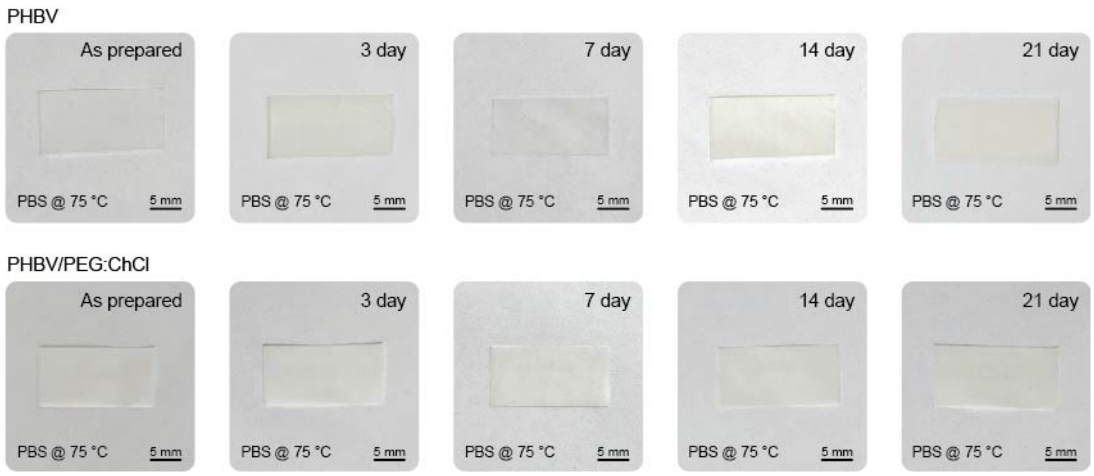

Supplementary Figure 16. **Transient performances of PHBV and PHBV/PEG:ChCl without HIU-triggering events.** Both membranes were immersed in PBS solution (pH 7.4, 75 °C) for 21 d, with the barely degraded structures representing their low transient rate.

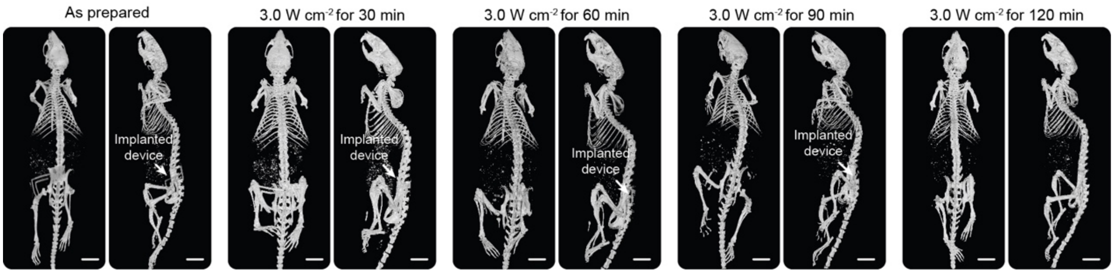

Supplementary Figure 17. **Micro-CT images captured at the top and side views.** The on-demand bioresorbable neurostimulator was fully disintegrated in 120 min upon the HIU events. The scale bar is equivalent to 10 mm.

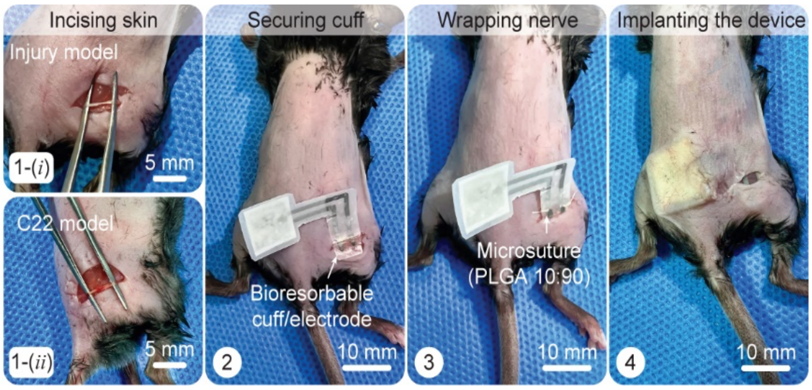

Supplementary Figure 18. **Surgical procedure to implant the on-demand bioresorbable neurostimulator.** The bioresorbable cuff electrode was wrapped around the sciatic nerve. Then, the ACT−TENG was implanted beneath the dermis.

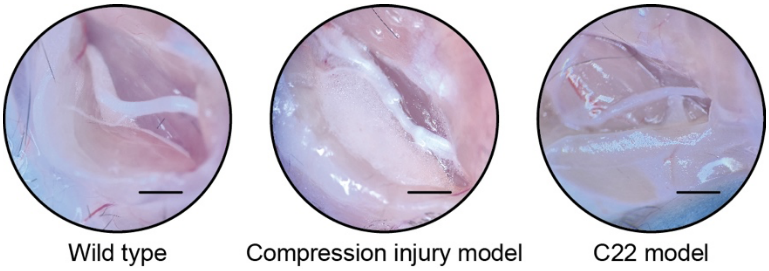

Supplementary Figure 19. **Magnified view of the sciatic nerve.** The images represent the nerve conditions of the wild type, compression injury model, and C22 model, respectively. We used a sterilized tissue retractor to secure the surgical site of the sciatic nerve by retracting adjacent muscle tissues. The scale bar is equivalent to 1 mm.

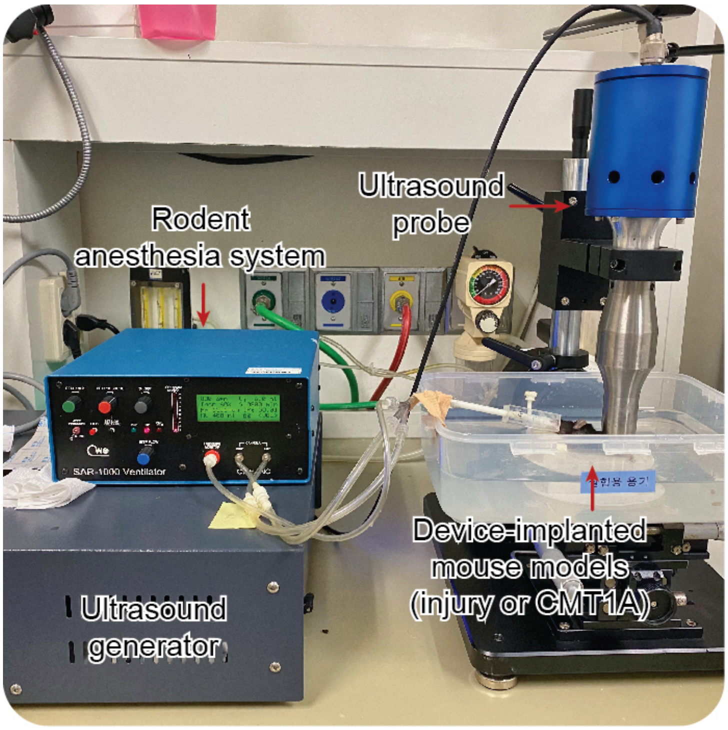

Supplementary Figure 20. **Experimental setup for the in vivo demonstration of peripheral nerve electrotherapy.** The device-implanted mouse was anesthetized during the ultrasound application time.

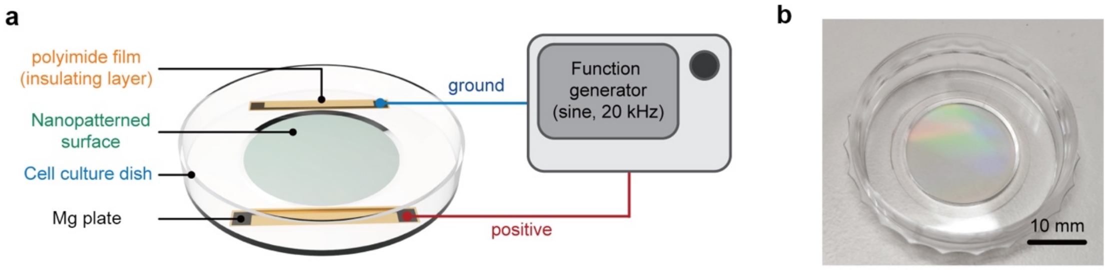

Supplementary Figure 21. **Experimental setup for in vitro demonstration of the iPSC-driven motor neuron growth upon electrical impulses. a** Schematic describing the experimental setup for the in vitro cell experiments. A pair of Mg plates (20 mm width, 2 mm length, 100 μm thickness) was attached to the nanopatterned cell culture dish to apply electrical impulses (20 kHz sinusoidal waveform). Insulating layers (polyimide films) were introduced to prevent short-circuit between metal electrodes and cell culturing media that may result in unwanted electrochemical reactions[3]. The iPSC-driven motor neuron cells were cultured on the nanopatterned surface to measure their axon length. **b** Real image of the nanopatterned cell culture dish.

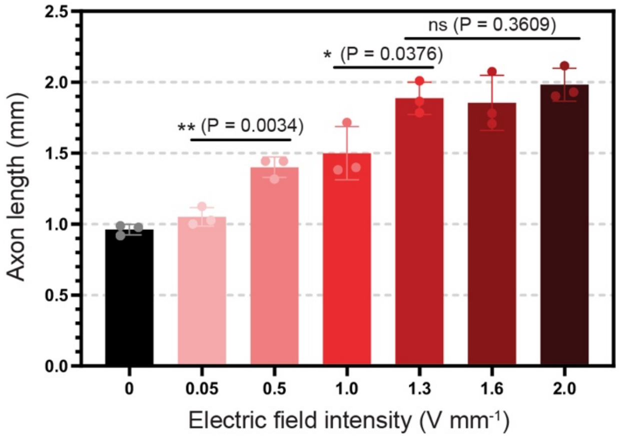

Supplementary Figure 22. **Axon length plots after 72 h of culturing time upon different electric field intensities.** The axon length exhibited saturated level at the 1.3 V mm^{-1} of electric field intensity, providing the electrical impulse condition for effective electrotherapy (n = 3 for each group). P values are evaluated through two-sided t test; ns = non significant; *P < 0.05; **P < 0.01.

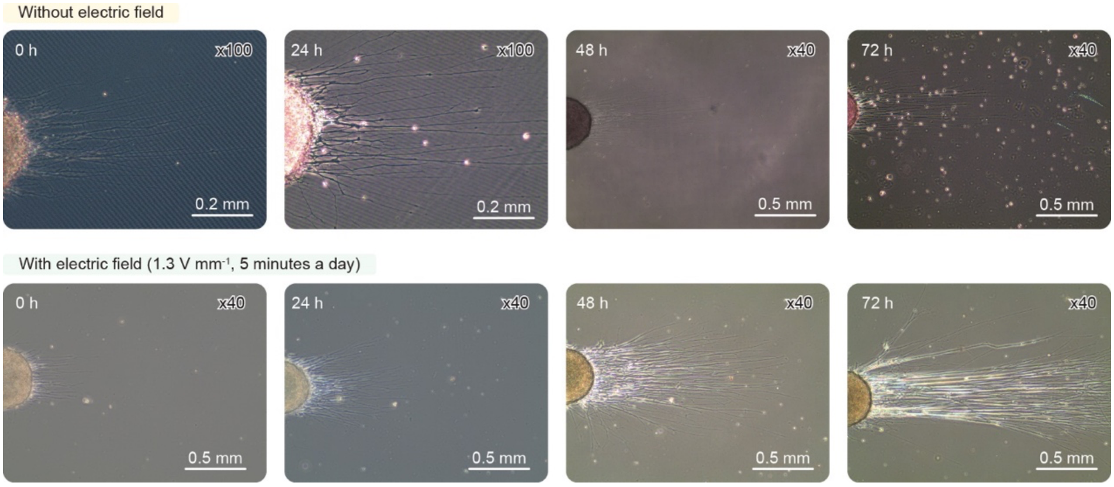

Supplementary Figure 23. **Optical microscopic images of iPSC-driven motor neuron.** The electrically treated iPSC-driven motor neuron cells exhibit longer axon length than the untreated cells.

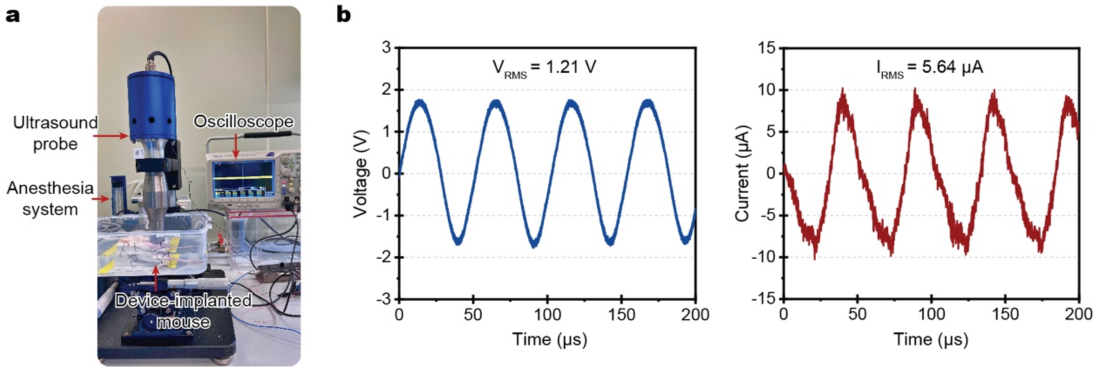

Supplementary Figure 24. **In vivo electrical characterization of the ACT−TENG. a** Experimental setup to characterize in vivo electrical output performances. **b** Triboelectric voltage at 40-megohm impedance, and **c** current at 1-ohm impedance by the ACT−TENG implanted in a mouse.

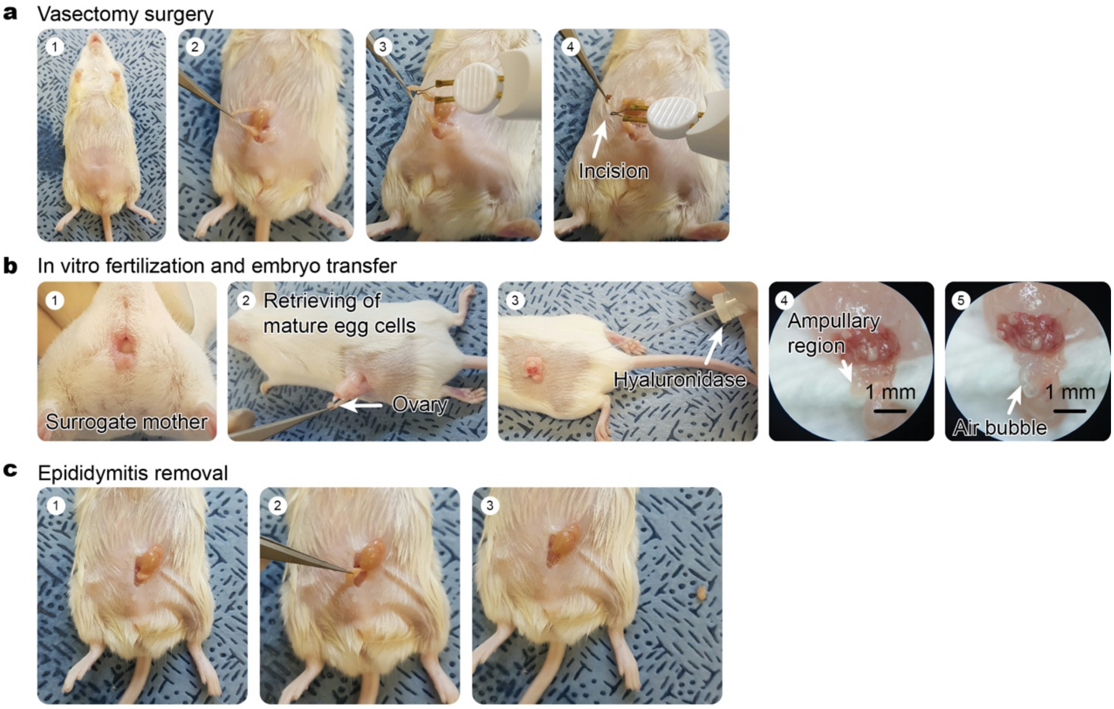

Supplementary Figure 25. **Detailed procedure for preparing C22 mouse model.** The preparation procedure of C22 mouse model can be divided into three steps: **a** vasectomy surgery, **b** in vitro fertilization with embryo transfer, and **c** epididymitis removal.

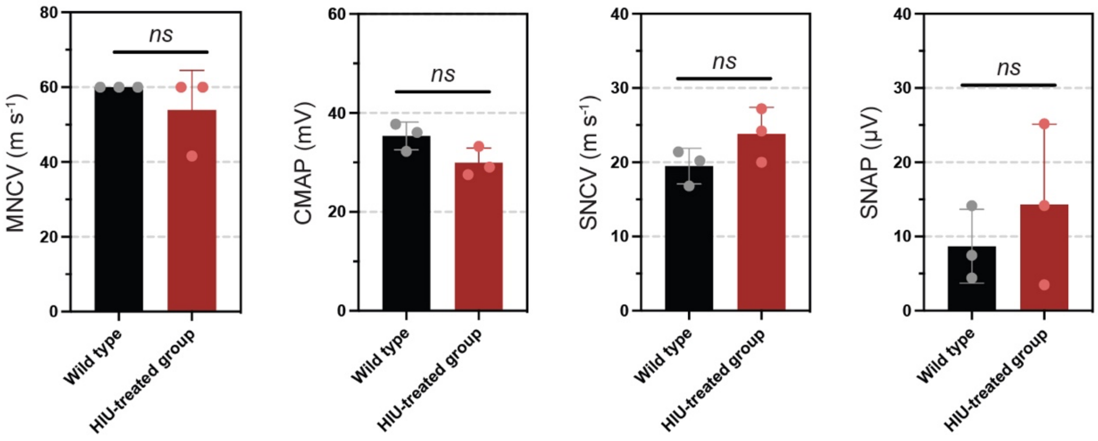

Supplementary Figure 26. **NCS results to evaluate the biosafety of the HIU to the sciatic nerve.** Here, the HIU-treated group represents the mouse model that encountered HIU treatments for 120 min (Wild type: n = 3. HIU-treated group: n = 3). All the parameters of the nerve conditions indicate that the HIU treatment does not induce any biological damage to the sciatic nerve. P values are evaluated through two-sided t test; ns = non significant (MNCV: P = 0.3739; CMAP: P = 0.0833; SNCV: P = 0.1583; SNAP: P = 0.4618).

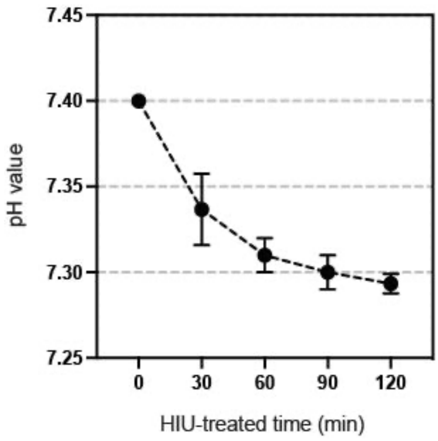

Supplementary Figure 27. **Characterization of pH change upon the HIU treatment.** PHBV membranes (n = 3) were immersed in diluted PBS solution (pH 7.4, 35 ºC). Upon the HIU treatment, the mechanically disintegrated PHBV membranes did not cause any significant change in pH of the diluted PBS solution.

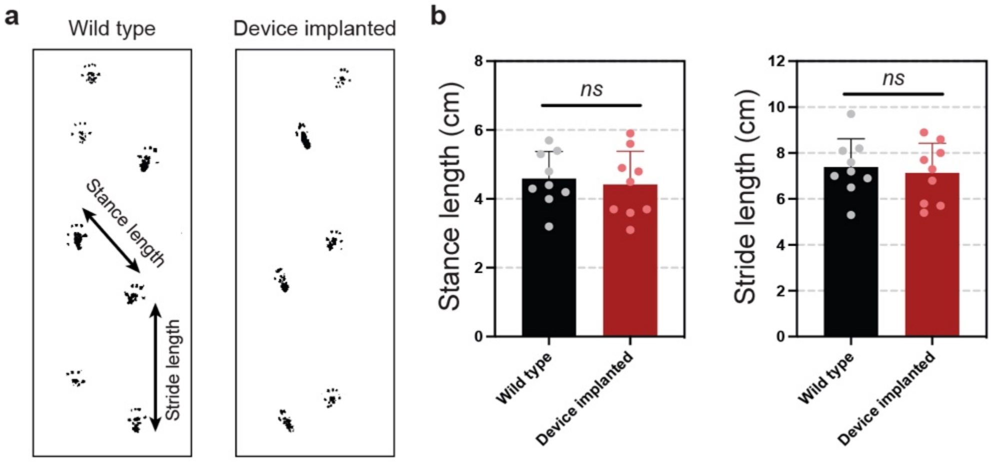

Supplementary Figure 28. **Behavior analysis of the device-implanted mouse.** To conduct footprint behavior analysis of wild type and device-implanted mouse, mice are trained to walk on a white paper before the trial. The pathway was constructed with acrylic walls and all paws of mice are painted with black ink (Supplementary Movie 6). Stride length is determined by measuring the distance between each step. **a** Footprints of the device-implanted mouse were obtained to verify if the device implantation causes any physical harm to the sciatic nerve. **b** The device-implanted mouse has comparable values of both stance length and stride length, the two main parameters for quantitative analysis of the footprints (Wild type: n = 9, Device implanted: n = 9). P values are evaluated through two-sided t test; ns = non significant (Stance length: P = 0.6933; Stride length: P = 0.6730).

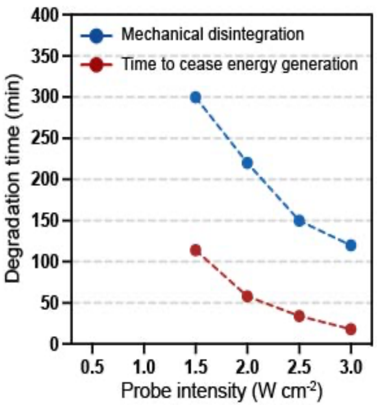

Supplementary Figure 29. **Characterization of degradation time at different ultrasound intensities.** The ACT−TENG was immersed in diluted PBS solution (pH 7.4, 25 °C) for electrical characterization. Then, we observed the following degradation time at different ultrasound intensities: i) time to cease energy generation, and ii) mechanical disintegration. Upon a low-intensity ultrasound (less than 1.0 W cm^{-2}), the ACT−TENG barely initiated its transient processes for several hours.

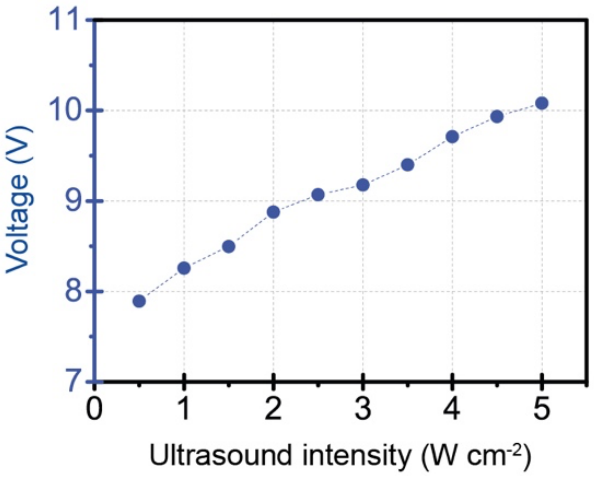

Supplementary Figure 30. **Ultrasound-driven triboelectric output performances of ACT-TENG at various ultrasound intensities (W cm^{-2}).**

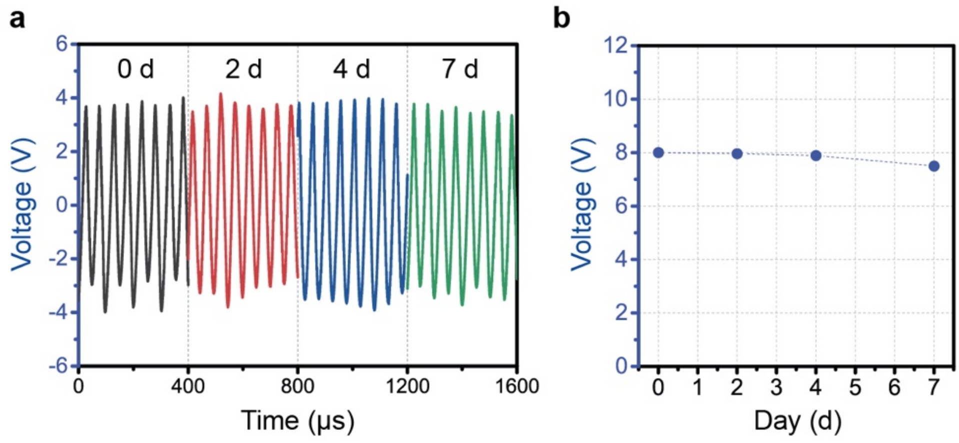

Supplementary Figure 31. **The ACT-TENG was immersed in PBS (pH 7.4, 37 °C) for 7 days.** **a** Ultrasound-driven triboelectric output performances depending on the days the device was immersed in PBS buffer solution. The voltage output remained similar during the experiment period. **b** Summation plots of Supplementary Figure 31a.

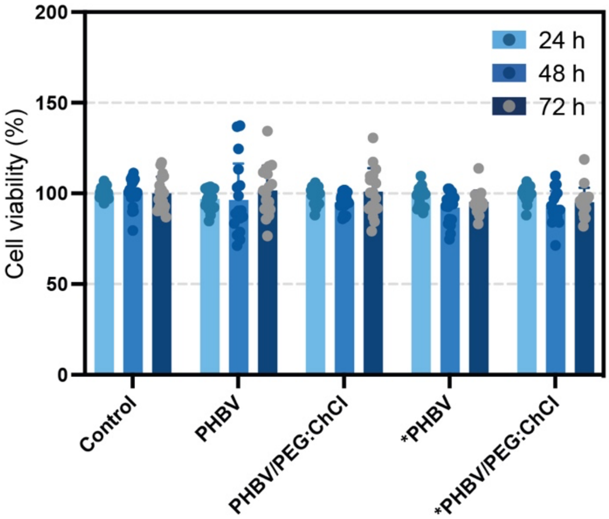

Supplementary Figure 32. **XTT cell proliferation assay results.** Since all the values are comparable to those of the control group, we confirmed cytotoxicity of the constituent polymers using XTT assay tests for 72 hours (n = 18 for each group). The asterisk mark (*) refers to the materials that were mechanically disintegrated by HIU triggering events.

Supplementary Note

Supplementary Note 1. Fundamental background of the localization of acoustic pressure inside the PHBV encapsulation layer

Acoustic impedance, defined as the ratio of the acoustic pressure to the sound velocity in a medium, provides a key to characterize the ultrasound transmission properties of materials[4]. Acoustic impedance can be derived from the Young's modulus and density of the medium, as shown below:

$$Z = \rho v = \sqrt{E \cdot \rho} \tag{1}$$

where, Z represents the acoustic impedance (unit: kg m^{-2} s^{-1}), v represents the sound of speed in a specific medium (unit: m s^{-1}), ρ represents the density of the medium (unit: kg m^{-3}), and E represents the Young's modulus of the medium (unit: kg m^{-1} s^{-2})[5].

As displayed in fig. S7 of the SI, we obtained the acoustic impedance value of the PHBV membrane through Eq. (1). The Young's modulus of the PHBV membrane (10 cm width, 1 cm length, 50 μm thickness) was measured using a universal testing machine (UTM) to identify its stress–strain curve. The density of the PHBV membrane was evaluated by a specific gravity scale. The measured Young's modulus and density were 7.34 GPa and 1,026.68 kg m^{-3}, respectively. Thus, we obtained the acoustic impedance of 2.75×10^6 for the PHBV encapsulation layer.

Due to the significant mismatch in the acoustic impedance between the PHBV membrane and the air medium (present in the porous structure of the PHBV, $Z_{air} = 0.0004 \times 10^6$ kg m^{-2} s^{-1}), reflection of the acoustic wave is dominant at the interface of the PHBV and the air[6]. The reflection coefficient can be calculated as follows:

$$a = \frac{(Z_2 - Z_1)^2}{(Z_2 + Z_1)^2} \tag{2}$$

where, a represents the reflection coefficient at the interface of material 1 and material 2, and Z_1 and Z_2 represent the acoustic impedance of material 1 and material 2, respectively. The reflection coefficient at the interface of the PHBV and the air was calculated to be 0.9994 based on Eq. (2), implying that 99.94 % of the acoustic wave is reflected. Therefore, most of the acoustic wave is reflected when it encounters the porous structure of the PHBV membrane, producing the localization of acoustic pressure field inside the PHBV porous structure.

Supplementary Note 2. Macroscopic degradation mechanism of PHBV

Previous studies have provided evidence of surface erosion behavior in various polyhydroxyalkanoates (PHAs), including PHBV, when exposed to physiological conditions[7,8]. This distinctive behavior is attributed to the inherent properties of PHBV, such as its low water permeability and hydrolysis reaction rate, making it apart from most bioresorbable polymers that undergo bulk erosion. These unique characteristics hold significant implications for ensuring the hermeticity of the device, thus influencing our choice of PHBV as the material for encapsulation layer.

First, the low water permeability of PHBV effectively prevents premature infiltration or leakage of liquid physiological media into the device. Moreover, the surface erosion behavior of PHBV serves as a protective mechanism, mitigating the risk of unexpected damage or severe mechanical disintegration of the encapsulation layer. By degrading gradually from the surface over an extended period, the PHBV ensures the stable device operation.

Supplementary References

1. Wang, X., Wang, S., Yang, Y. & Wang, Z. L. Hybridized electromagnetic-triboelectric nanogenerator for scavenging air-flow energy to sustainably power temperature sensors. *ACS Nano* **9**, 4553-4562 (2015).
2. Sato, H., Murakami, R., Padermshoke, A., Hirose, F., Senda, K., Noda, I. & Ozaki, Y. Infrared spectroscopy studies of CH···O hydrogen bondings and thermal behavior of biodegradable poly(hydroxyalkanoate). *Macromol.* **37**, 7203-7213 (2004).
3. Kirson, E.D., Gurvich, Z., Schneiderman, R., Dekel, E., Itzhaki, A., Wasserman, Y., Schatzberger, R. & Palti, Y. Disruption of cancer cell replication by alternating electric fields. *Cancer Res.* **64**, 3288-3295 (2004).
4. Regtien, P. P. L. *Sensors for Mechatronics* (Elsevier, Amsterdam, Netherlands, 2012).
5. Mattoon, J. S. & Nyland, T. G. *Small Animal Diagnostic Ultrasound* (Elsevier Saunders, St. Louis, MO, 2020).
6. Gomez, T. E. & Montero, F. Bridging the gap of impedance mismatch between air and solid materials. *IEEE Int. Ultrason. Symp.* **2**, 1069-1072 (2000).
7. Choi, S. Y., Cho, I. J., Lee, Y., Kim, Y.-J., Kim, K.-J. & Lee, S. Y. Microbial polyhydroxyalkanoates and nonnatural polyesters. *Adv. Mater.* **32**, 1907138 (2020).
8. Salomez, M., George, M., Fabre, P., Touchaleaume, F., Cesar, G., Lajarrige, A. & Gastaldi, E. A comparative study of degradation mechanisms of PHBV and PBSA under laboratory-scale composting conditions. *Polym. Degrad. Stab.* **167**, 102-113 (2019).
