# Supplementary material for: An on-demand bioresorbable neurostimulator
Source: Nat Commun. 2023 Nov 11;14:7315. doi: 10.1038/s41467-023-42791-5 (PMC10640647; doi:10.1038/s41467-023-42791-5)
Supplement: Supplementary file 3 — Description of Additional Supplementary Files [file 41467_2023_42791_MOESM3_ESM.pdf]

<u>Description of additional supplementary files</u>

Supplementary Movie 1. In vitro ultrasound-driven triboelectric output performance.

Supplementary Movie 2. In vitro HIU-triggered on-demand transient process.

Supplementary Movie 3. In vivo ultrasound-driven triboelectric output performance.

Supplementary Movie 4. In vivo ultrasound-driven triboelectric output performance at different implant locations.

Supplementary Movie 5. Nerve conduction study (NCS) experiments: Methods.

Supplementary Movie 6. Footprint analysis to verify the biosafety of device implantation.
