# Supplementary material for: An on-demand bioresorbable neurostimulator
Source: Nat Commun. 2023 Nov 11;14:7315. doi: 10.1038/s41467-023-42791-5 (PMC10640647; doi:10.1038/s41467-023-42791-5)
Supplement: Supplementary file 10 — Reporting Summary [file 41467_2023_42791_MOESM10_ESM.pdf]

Corresponding author(s): Byung-Ok Choi, Sang-Woo Kim

Last updated by author(s): Oct 9, 2023

Reporting Summary

Nature Portfolio wishes to improve the reproducibility of the work that we publish. This form provides structure for consistency and transparency in reporting. For further information on Nature Portfolio policies, see our Editorial Policies and the Editorial Policy Checklist.

Statistics

For all statistical analyses, confirm that the following items are present in the figure legend, table legend, main text, or Methods section.

n/a	Confirmed	
☐	☒	The exact sample size (*n*) for each experimental group/condition, given as a discrete number and unit of measurement
☐	☒	A statement on whether measurements were taken from distinct samples or whether the same sample was measured repeatedly
☐	☒	The statistical test(s) used AND whether they are one- or two-sided<br>*Only common tests should be described solely by name; describe more complex techniques in the Methods section.*
☒	☐	A description of all covariates tested
☒	☐	A description of any assumptions or corrections, such as tests of normality and adjustment for multiple comparisons
☐	☒	A full description of the statistical parameters including central tendency (e.g. means) or other basic estimates (e.g. regression coefficient) AND variation (e.g. standard deviation) or associated estimates of uncertainty (e.g. confidence intervals)
☐	☒	For null hypothesis testing, the test statistic (e.g. *F*, *t*, *r*) with confidence intervals, effect sizes, degrees of freedom and *P* value noted<br>*Give P values as exact values whenever suitable.*
☒	☐	For Bayesian analysis, information on the choice of priors and Markov chain Monte Carlo settings
☒	☐	For hierarchical and complex designs, identification of the appropriate level for tests and full reporting of outcomes
☒	☐	Estimates of effect sizes (e.g. Cohen's *d*, Pearson's *r*), indicating how they were calculated

Our web collection on statistics for biologists contains articles on many of the points above.

Software and code

Policy information about availability of computer code

Data collection	Electrical characterization data were collected using Tektronix oscilloscope (Tektronix, DPO3052) data saving software. Micro-CT images were taken using Skyscan 1276 (Bruker).
Data analysis	Electrical characterization data were analyzed using OriginPro 9.0 and Microsoft Excel (Version 16.71, 23031200). Material characterization data were analyzed using OriginPro 9.0 and Microsoft Excel (Version16.71, 23031200). FEM simulations performed in this work were carried out using COMSOL Multiphysics (Version 5.5). All the statistical analysis were conducted using Prism 9 software (Version 9.5.0, GraphPad). The following softwares were used to conduct micro-CT image processing: NRecon (Bruker) for the reconstruction process, CTAn software (Bruker) for the analysis of reconstructed images, and CTVox software (Bruker) for the 3D-rendering process. The axon length of the nervous tissue was measured using ZEISS ZEN (blue edition) to perform histopathological studies.

For manuscripts utilizing custom algorithms or software that are central to the research but not yet described in published literature, software must be made available to editors and reviewers. We strongly encourage code deposition in a community repository (e.g. GitHub). See the Nature Portfolio guidelines for submitting code & software for further information.

Data

Policy information about availability of data

All manuscripts must include a data availability statement. This statement should provide the following information, where applicable:

- Accession codes, unique identifiers, or web links for publicly available datasets
- A description of any restrictions on data availability
- For clinical datasets or third party data, please ensure that the statement adheres to our policy

The data generated in this study are provided in the Supplementary Information/Source Data file. The data used in this study are available in the Figshare Repository under accession code [https://doi.org/10.6084/m9.figshare.22560232].

Research involving human participants, their data, or biological material

Policy information about studies with human participants or human data. See also policy information about sex, gender (identity/presentation), and sexual orientation and race, ethnicity and racism.

Reporting on sex and gender	We obtained informed consent from a single female participant for this research. We also acquired consent to publish information that identifies this individual.
Reporting on race, ethnicity, or other socially relevant groupings	For human iPSC preparation, we collected skin tissues to procure fibroblasts from an Asian participant.
Population characteristics	The participant mentioned above is 53-year-old.
Recruitment	We conducted recruitment of the participant based on the Helsinki Declaration and the Institutional Review Boards (IRB) of Samsung Medical Center at Sungkyunkwan University (2021-04-053-001).
Ethics oversight	Samsung Medical Center at Sungkyunkwan University approved the study protocol.

Note that full information on the approval of the study protocol must also be provided in the manuscript.

Field-specific reporting

Please select the one below that is the best fit for your research. If you are not sure, read the appropriate sections before making your selection.

☒ Life sciences ☐ Behavioural & social sciences ☐ Ecological, evolutionary & environmental sciences

For a reference copy of the document with all sections, see nature.com/documents/nr-reporting-summary-flat.pdf

Life sciences study design

All studies must disclose on these points even when the disclosure is negative.

Sample size	In order to conduct pre-clinical studies, we aimed to fabricate our ACT-TENG with minimal dimension. Our electrical characterization and in vitro experiment results confirmed that our ACT-TENG with 1 cm x 1 cm dimension is sufficient to stimulate the lesion of the sciatic nerve.
Data exclusions	No data were excluded from the analyses.
Replication	H&E-stained images: In order to validate the inflammatory response throughout the biodegradation process, five mice for each operation mechanism were sacrificed, one at a time during each designated time period, to obtain H&E-stained tissue images<br>Micro-CT images: Our bioresorbable neurostimulator was implanted into a mouse to confirm the in vivo demonstration of the ultrasound-mediated transience.<br>Pre-clinical studies of the compression peripheral nerve injury mouse model: Our bioresorbable neurostimulators were implanted into 5 mice for each group to validate therapeutic effects of the electrical stimulation events through nerve conduction studies and histopathological studies.<br>Pre-clinical studies of the hereditary peripheral neuropathy (CMT1A) mouse model: Our bioresorbable neurostimulators were implanted into 5 mice for each group to validate therapeutic effects of the electrical stimulation events through nerve conduction studies and histopathological studies.<br>All the experiments above, regarding life sciences study, were successfully replicated unless there were any technical issues.
Randomization	Our mouse models (10 weeks, C57BL/6) were randomly chosen regardless of their conditions (sex, body weight, etc.).
Blinding	Blinding was not applicable in this study as the device is entirely isolated, rendering a blinding procedure irrelevant to the outcomes.

Reporting for specific materials, systems and methods

We require information from authors about some types of materials, experimental systems and methods used in many studies. Here, indicate whether each material, system or method listed is relevant to your study. If you are not sure if a list item applies to your research, read the appropriate section before selecting a response.

Materials & experimental systems

n/a	Involved in the study
[x]	[ ] Antibodies
[ ]	[x] Eukaryotic cell lines
[x]	[ ] Palaeontology and archaeology
[ ]	[x] Animals and other organisms
[x]	[ ] Clinical data
[x]	[ ] Dual use research of concern
[x]	[ ] Plants

Methods

n/a	Involved in the study
[x]	[ ] ChIP-seq
[x]	[ ] Flow cytometry
[x]	[ ] MRI-based neuroimaging

Eukaryotic cell lines

Policy information about cell lines and Sex and Gender in Research

Cell line source(s)	Human fibroblasts (ATCC, CRL-1502) were used in this study (Designation: WS1; Product category: Human cells; Organism: Homo sapience, human; Cell type: Fibroblast; Morphology: Fibroblast).
Authentication	The human fibroblasts purchased through ATCC were not authenticated.
Mycoplasma contamination	The human fibroblasts (ATCC, CRL-1502) were tested negative for mycoplasma contamination.
Commonly misidentified lines (See ICLAC register)	No commonly misidentified cell lines were used in this work.

Animals and other research organisms

Policy information about studies involving animals; ARRIVE guidelines recommended for reporting animal research, and Sex and Gender in Research

Laboratory animals	The 10-week-old C57BL/6J and CrlOri:CD1(ICR) females were prepared and provided with a standard diet and water ad libitum, and housed in a temperature-controlled (22 ± 2) ℃ and humidity-controlled (44–56) % environment with a 12 h light–dark cycle.<br>The C22 mice [B6; CBACa-Tg (PMP22) C22Clh/H], which harbor seven copies of a human PMP22 transgene (Huxley et al., 1996), were obtained from MRC Harwell (Oxfordshire, UK; IMSR Cat# HAR:784, RRID: IMSR_HAR:784). The C22 and wild type (WT) mice were produced by in vitro fertilization using 10–week–old C57BL/6J females (OrientBio, Republic of Korea) and 12–week–old C22 males (Macrogen, RRID:SCR_014454). Animals were provided with a standard diet and water ad libitum, and housed in a temperature-controlled (22 ± 2) ℃ and humidity-controlled (44–56) % environment with 12 h light–dark cycle. Male C22 or WT littermates were used for experiments when they were three weeks old.
Wild animals	The study did not involve wild animals.
Reporting on sex	Thirty C57BL/6J and twenty CrlOri:CD1(ICR) mice were all females.<br>Thirty C22 mice were made of 20 females and 10 males.
Field-collected samples	The study did not involve field-collected samples.
Ethics oversight	All procedures including electrical characterization, surgical processes, in vivo demonstration, and biosignal monitoring of the experimental subjects were performed according to protocols approved by the Institutional Animal Care and Use Committee (IACUC) of Samsung Medical Center (SMC, 20210318001).

Note that full information on the approval of the study protocol must also be provided in the manuscript.
