# Supplementary material for: An on-demand bioresorbable neurostimulator
Source: Nat Commun. 2023 Nov 11;14:7315. doi: 10.1038/s41467-023-42791-5 (PMC10640647; doi:10.1038/s41467-023-42791-5)
Supplement: Supplementary file 11 — Source Data [file 41467_2023_42791_MOESM11_ESM.zip › Figure 4/Fig. 4a/WT semithin.pdf]

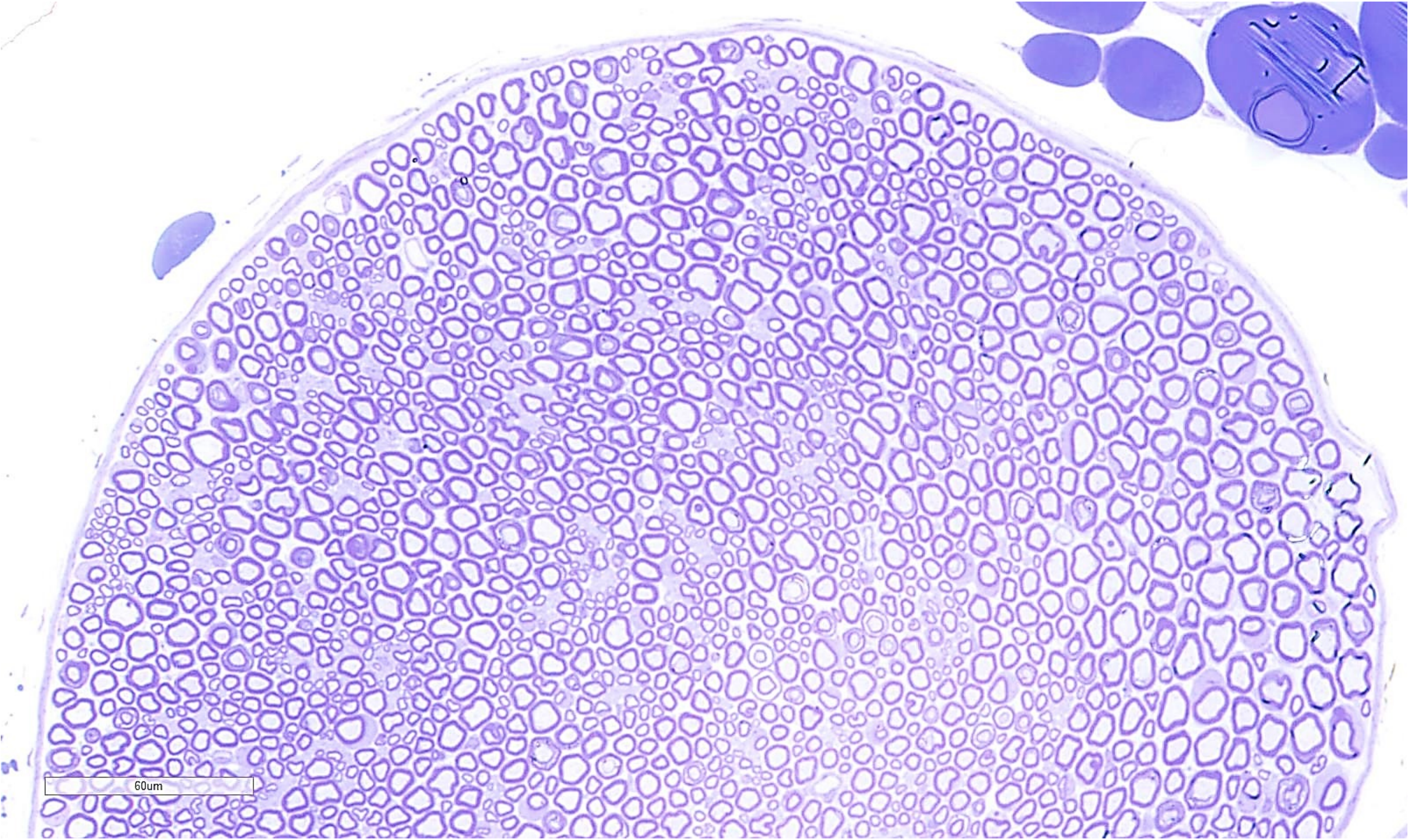
60um
